# Adenomatoid odontogenic tumor associated to reactive fibro-osseous lesion: A case report

**DOI:** 10.4317/jced.60184

**Published:** 2023-08-01

**Authors:** René Jara, Paz Martínez, Victoria Rees, Benjamín Martínez, Sergio González-Providell

**Affiliations:** 1 DDS, MSc, Oral Pathology, Dental School, Faculty of Medicine and Health Sciences, Mayor University, Santiago, Chile; 2 DDS, Private practice; 3 DDS, Post-graduate student, Oral Pathology, Dental School, Faculty of Medicine and Health Sciences, Mayor University, Santiago, Chile; 4 DDS, MSc, Full professor, Oral Pathology, Dental School, Faculty of Medicine and Health Sciences, Mayor University, Santiago, Chile; 5 DDS, MSc, Full professor, Oral Pathology, Dental School, Faculty of Medicine and Health Sciences, Mayor University, Santiago, Chile. Oral Pathology Department, Faculty of Dentistry, Universidad de los Andes, Santiago, Chile

## Abstract

An adenomatoid odontogenic tumor (AOT) is a benign epithelial lesion, being the fourth most common among all odontogenic tumors. Usually presents as slow painless growth that sometimes leads to facial asymmetry. Many cases are detected by radiographic studies, and the indication for biopsy and surgery is secondary to this finding. We report a case of a 17-year-old man with a history of left mandibular painless swelling since 4 months ago, associated with facial asymmetry and hard consistency. An imaging study showed an extensive unilocular radiolucent lesion to the basilar arch, with defined limits and with peripheral hyperdense areas located only vestibular to the lesion. The histopathology was composed of odontogenic epithelial cell proliferation, with epithelial nodular and duct-like structures, rosettes of spindled epithelial cells with eosinophilic material, calcifications, and fibro-osseous reaction. Surgical conservative excision including the affected tooth is the treatment of choice and recurrence is rare. The histologic findings of reactive fibro-osseous proliferation in AOT should be interpreted as a reactive change in the tumor capsule and not as an adnexal lesion. We present an atypical case of AOT with reactive fibro-osseous reaction. Despite clinical aggressive behavior, conservative surgical treatment could be the treatment of choice. Additionally, we emphasize the importance of histopathological examination together with the imaging study of radiolucent lesions of the maxillary bones.

** Key words:**Adenomatoid Odontogenic Tumor, Odontogenic tumor, fibro-osseous lesion.

## Introduction

Adenomatoid odontogenic tumor (AOT) is a benign epithelial lesion, being the fourth most common among of all odontogenic tumors (1), representing 3% of them (2). Odontogenic epithelium is the main histological feature, with several patterns(3).

This lesion has been named in several ways: epithelial odontoma, cystic epithelial tumor, adamantinoma, mandibular tooth germ cyst, development cyst associated epithelial tumor, adenoameloblastoma, ameloblastic adenomatoid tumor (4). Finally, was given the name adenomatoid odontogenic tumor, proposed by Philipsen and Birn, and accepted by the World Health Organization classification of odontogenic tumors in 1997 (2).

There are different points of views in the literature regarding if it is a neoplastic or hamartomatous lesion (5). In previous classifications, it was classified as a mixed odontogenic tumors, considering the mixed degrees of inductive changes. Nevertheless, the current WHO classification stands that the presence of calcifications inside AOT, was not due to induction, but because of a mineralization metaplastically developed, and thus, the AOT was reclassified into the odontogenic epithelium derived group (3,6).

The age of presentation of AOT is between 3 to 82 years (Naidu *et al*. 4), however, 88% of cases are diagnosed in the second and third decades of life (Rick 8). Gender and anatomical site, apparently are related to age of occurrence; before 30 years of age maxillary anterior site is more frequently affected. After 30 years of age, mandibles are more frequently affected, and males more than females; although AOT generally has a 2:1 female to male ratio (4).

It’s a firm, slow growing swelling; may expand the cortical plates, and lead to face asymmetry. Generally is pain free, so the patient seeks professional care very late, and so an important growth is detecTable at that time (7). Most AOT are less than 3 cm in diameter (8). Three clinical presentations of AOT are recognized: follicular (70,8%), with an intraosseous growth associated with a nonerupted tooth; extrafollicular (26,9%) not associated with the tooth crown, but in intraosseous position; and the peripheral (2,3%). These three variants respond in every way to a benign lesion (9).

A high number of AOT cases are detected by radiographic studies, since over 95% are of intraosseous origin. It appears as a well-defined corticalized unilocular radiolucency. Approximately 71% of all AOT are associated with the crown of an unerupted tooth, ranging from 40% to 60% the upper canines (8), many times being confused with a dentigerous cyst. In the inside of the lesion, a variable shape and size of radiopacities are possible to be observed, whether scattered or grouped. Frequently this lesion modifies the associated tooth position, and large tumors can produces root resorption (8).

Histopathologically is composed of an odontogenic epithelial cell proliferation, forming epithelial nodular and duct-like structures, rosettes of spindled epithelial cells with eosinophilic material and calcifications. Additionally, it shows a fibrous capsule (8).

Surgical conservative excision including the affected tooth is the treatment of choice and recurrence is rare (1).

The purpose of this article is to present an atypical case of AOT, rarely seen, and to review the literature of the lesion.

## Case Report

A 17-year-old man consulted for a mandibular swelling since 4 months ago, without associated symptoms. He had no relevant medical history. He did not reported toxic habits or important family history.

**Figure 1:**
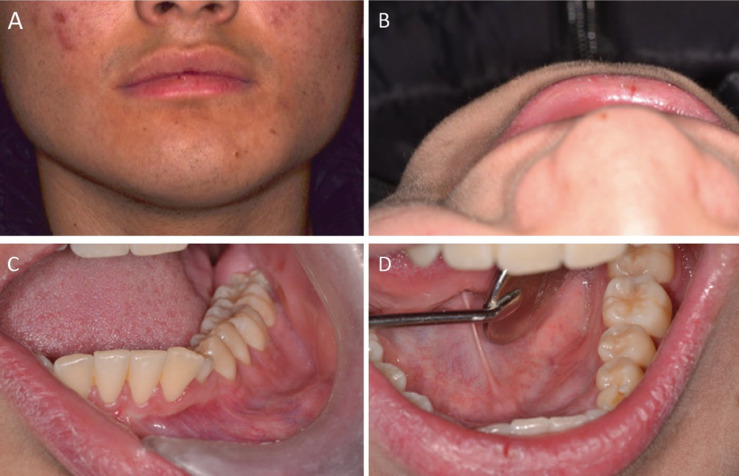
Extra and intraoral examination. A and B, Extraoral clinical examination, the skin covering the area was unaltered (A), facial asymmetry due to increased volume in the left mandibular region (B). C and D Intraoral clinical examination, vestibular enlargement of teeth 7.3, 3.4 and 3.5, covered by healthy oral mucosa. There was persistence of tooth 7.3 and absence of tooth 3.3 (C). Lingual enlargement covered by healthy oral mucosa (D).

The extraoral clinical examination revealed facial asymmetry due to swelling in the left mandibular region, of hard consistency, immobile and painless. The skin covering the area was without alterations. (Fig. 1A,B).

The intraoral clinical examination showed a lingual and vestibular swelling in relation to teeth 7.3, 3.4 and 3.5, of hard consistency and covered by healthy oral mucosa. There was persistence of tooth 7.3 and absence of tooth 3.3 (Fig. 1C,D). The related teeth were vital.

**Figure 2:**
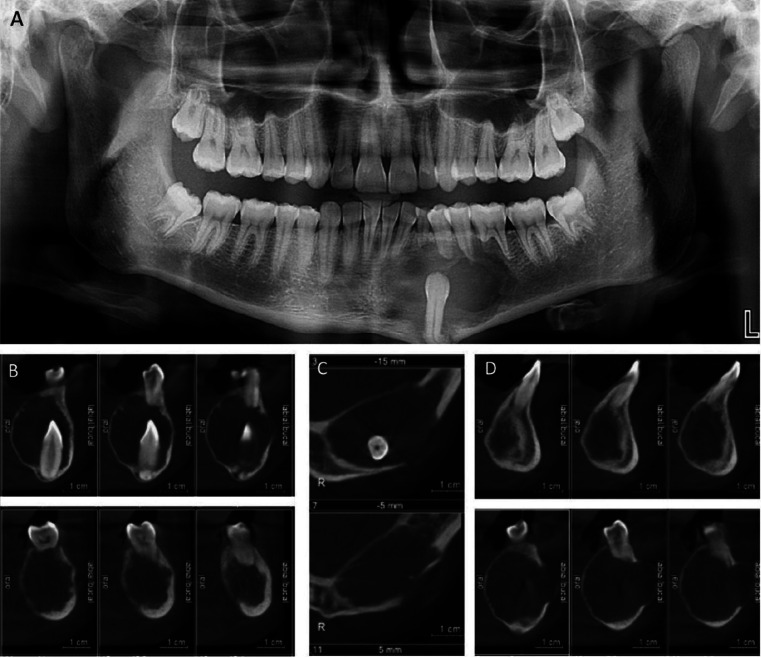
Panoramic X-ray and Cone beam computed tomography. A, Panoramic X-ray, extensive unilocular radiolucent lesion surrounding crown and root of tooth 3.3. It causes bulging of the basilar border, root resorption of teeth 7.3, 3.4, 3.5, 3.6 and dental displacement of tooth 3.1 and 3.2. Unerupted tooth 3.3 included in vertical position and displaced to basilar. B to D, Cone beam computed tomography, oval-shaped hypodense lesion causing marked root resorption of tooth 3.6 (B). Hypodense lesion causing expansion and thinning of vestibular and lingual tables, with perforation of the former (C). Oval-shaped hypodense lesion with peripheral hyperdense area located only vestibular to the lesion (D).

The imaging study included a panoramic radiograph and cone beam computed tomography. The panoramic radiograph showed an extensive unilocular radiolucent lesion, with defined limits at the level of the left mandibular body, surrounding the crown and root of tooth 3.3, with a large radiolucency area distal to the impacted tooth.. It extends mesio-distally from the apical region of teeth 3.1 and 3.2 to the distal apical area of tooth 3.6 and in the caudal cranial extending from the area close to the marginal bone crest to the mandibular basilar border area. It causes bulging of the basilar edge, root resorption of teeth 7.3, 3.4, 3.5, 3.6 and dental displacement of tooth 3.1 and 3.2. Unerupted tooth 3.3 in vertical position displaced to basilar, root curved and projected on basilar edge (Fig. 2A).

The cone beam computed tomography showed an oval-shaped hypodense lesion of approximately 37 mm in the mesiodistal direction, 24 mm in the vestibulolingual direction and 30 mm in the cephalocaudal direction that produced expansion and thinning of the vestibular and lingual Tables, with perforation of the first one, displacement of the mandibular canal to the vestibular and marked root resorption of tooth 3.6. In addition, a peripheral hyperdense area located only on the vestibular side of the lesion (Fig. 2B-D).

**Figure 3:**
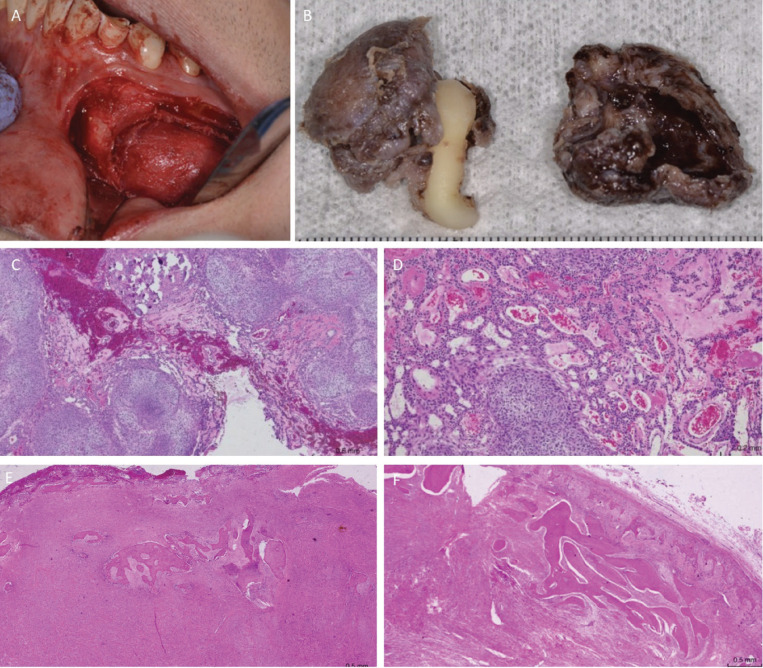
Intraoperative, macroscopic and microscopic features of the lesion. A, aspect after vestibular flap. B, surgical specimen with tooth and thick capsule. C and D, Epithelial proliferation with whorled areas and areas similar to ducts (Hematoxylin-Eosin, original magnification 20X). E and F, large areas of fibroblastic proliferation with bone trabeculae. (Hematoxylin - Eosin, original magnification 20X).

Based on the clinical and radiographic findings, the diagnostic hypothesis was follicular adenomatoid odontogenic tumor. Excisional biopsy was performed, which allowed excision of the lesion and extraction of tooth 3.3 (Fig. 3A).

Macroscopically, the sample consisted of two tissue fragments; the first, a firm brownish cystic membrane, which surrounded the crown and partially the root of the left canine (3.3), whose size was 30x22x15mm; and the second, a thick cystic like membrane of 30x25x15mm, brownish color, which was firm when cut (Fig. 3B). Both fragments were submitted to histopathological study.

Microscopically, the first fragment was composed of a cystic like membrane whose lining presented a nodular and solid proliferation of elongated odontogenic epithelial cells, forming duct-like and other rosette-like structures, with central eosinophilic material. The cystic wall was formed by abundant fibrocellular connective tissue. (Fig. 3C,D).

In the second fragment, the specimen was composed of a capsule of fibrous tissue and a proliferation of fused cells forming thick trabeculae of calcified eosinophilic cement-like extracellular matrix (Fig. 3E,F).

Based on the histomorphologic findings mentioned above, a diagnosis of adenomatoid odontogenic tumor with peripheral fibro-osseous reaction was made.

## Discussion

Our case is an atypical presentation of a follicular-type adenomatoid odontogenic tumor associated with a fibro-osseous reaction. To date there are only two reports in the literature, describing three similar cases (10,4).

Adenomatoid odontogenic tumors have a female predilection and are frequently located in the maxilla (8,4). The three cases previously reported in the literature that presented an associated fibro-osseous reaction (10,4), corresponded to lesions located in maxilla and in females, coinciding with the classic clinical features of AOT, unlike our case, male and mandible.

The two cases described by Naidu *et al*. and ours correspond to the follicular variant, with an unerupted canine. The case of Li *et al*. corresponded to the extrafollicular variant.

Similar to the AOT described in the multicenter study by Luiza *et al*., our patient presented with an asymptomatic lesion, causing facial asymmetry as a result of cortical bone expansion with diffuse vestibular and lingual swelling. None of the AOTs presented in that study showed association with an adjacent fibro-osseous reaction unlike our case and those described by Li *et al*. in 2013 and Naidu *et al*. in 2016 (10,4).

Intraosseous AOT (follicular and extrafollicular), almost invariably show imaging characteristics of a benign odontogenic lesion such as a well-demarcated radiolucency may present radiopacities, almost always unilocular, regular edge, pericoronary or juxtacoronary location and an average diameter between 1 to 3 cm. (Rick 8). Interestingly, in our case more aggressive radiographic features than usual were observed, such as the presence of root resorption, thinning and perforation of bone Tables and bulging of the basilar border.

The AOT reported by Li *et al*. also presented as a unilocular radiolucent lesion with resorption of the apices of the involved teeth. But the cases reported by Naidu *et al*. were described as mixed lesions, due to the presence of calcifications in the center of the lesion, without root resorption of the related teeth and with poorly defined limits.

Histopathologically, as in the cases reported by Naidu *et al*. and Li *et al*. the fibro-osseous proliferation enveloped the AOT peripherally as a shell. Both papers, interpret this feature as a secondary reactive proliferation in the capsule of the AOT.

Enucleation of the lesion is the appropriate treatment for these cases with close follow-up including clinical and radiographic examinations (9). It is not reported modification in the treatment of AOT with fibro-osseous proliferation, although this feature allowed an easily enucleation in our case.

The nature of AOT is controversial, and it is considered to be either neoplastic or hamartomatous. Studies based on its clinical behavior, such as its small size in most cases, minimal potential growth and no recurrence, radiographic features, and the absence of true neoplastic behavior and malignant transformation, suggest a rather hamartomatous nature (9). However, it should be noted that the small size of AOT would be due to the fact that most are detected early (on a routine dental radiograph) and removed before the tumor reaches a clinically noticeable size (8), unlike the reported case, in which the patient consults because of the swelling and not because of the absence of tooth 3.3 and persistence of tooth 7.3.

Other studies (11,12) suggest a neoplastic nature, related to the existence of a mutation in the KRAS proto-oncogene, which is frequently mutated in lung cancer, pancreatic and colorectal adenocarcinomas, and which has currently been detected in a high percentage of adenomatoid odontogenic tumors.

According to Coura *et al*. no association was found between the presence of the KRAS proto-oncogene mutation and the clinical characteristics of AOT, such as lesion size, location, clinical variants, patient age and capsule thickness. The mutation would not be conditioning for lower or higher tumor aggressiveness (11).

In addition, the activation of the MAPK/ERK pathway has been reported, evidenced by the immunohistochemical expression of proteins of this pathway such as EGFR, KRAS, BRAF, among others, in AOT epithelial cells. However, EGFR expression is not exclusive to AOT, and is also observed in the odontogenic epithelium of dental germs and epithelial debris, suggesting that its expression is regulated during odontogenesis. Some EGFR patterns have been linked to the potential to develop various odontogenic cysts and tumors such as radicular cysts, dentigerous cysts, keratocysts, ameloblastomas, and ameloblastic fibromas (11,12).

## Conclusions

AOT is a benign odontogenic tumor that can have different clinical presentations. The reported case corresponds to a AOT associated with a peripheral fibro-osseous reaction, similar to the cases of Naidu *et al*. and Li *et al*.

Unlike conventionally described AOT, the behavior of the lesion was more aggressive, presenting root resorption, expansion and perforation of slabs, unlike conventionally described AOT, similar to that reported by Naidu *et al*. and Li *et al*.

The histologic findings of reactive fibro-osseous proliferation in AOT should be interpreted as reactive changes in the tumor capsule and not as an adnexal lesion. Despite the histological findings reported, the conservative surgical treatment could be the treatment of choice for this type of tumor, maintaining close follow-up, including clinical and imaging examinations.

We emphasize the importance of histopathological examination together with the imaging study of radiolucent lesions of the maxillary bones.
